# Cross-Linked Cyclodextrins Bimetallic Nanocatalysts: Applications in Microwave-Assisted Reductive Aminations

**DOI:** 10.3390/molecules25020410

**Published:** 2020-01-19

**Authors:** Emanuela Calcio Gaudino, Elisa Acciardo, Silvia Tabasso, Maela Manzoli, Giancarlo Cravotto, Rajender S. Varma

**Affiliations:** 1 Dipartimento di Scienza e Tecnologia del Farmaco and NIS—Centre for Nanostructured Interfaces and Surfaces, University of Turin, Via Giuria 9, 10125 Turin, Italy; emanuela.calcio@unito.it (E.C.G.); elisa.acciardo@gmail.com (E.A.); maela.manzoli@unito.it (M.M.); 2 Dipartimento di Chimica, University of Turin, Via P. Giuria 7, 10125 Turin, Italy; silvia.tabasso@unito.it; 3 Regional Centre of Advanced Technologies and Materials, Department of Physical Chemistry, Faculty of Science, Palacky University, Šlechtitelů 27, 783 71 Olomouc, Czech Republic; varma.rajender@epa.gov

**Keywords:** heterogeneous catalysis, bimetallic nanocatalyst, microwaves, one-pot reductive amination, sustainable protocols

## Abstract

The optimization of sustainable protocols for reductive amination has been a lingering challenge in green synthesis. In this context, a comparative study of different metal-loaded cross-linked cyclodextrins (CDs) were examined for the microwave (MW)-assisted reductive amination of aldehydes and ketones using either H_2_ or formic acid as a hydrogen source. The Pd/Cu heterogeneous nanocatalyst based on Pd (II) and Cu (I) salts embedded in a β-CD network was the most efficient in terms of yield and selectivity attained. In addition, the polymeric cross-linking avoided metal leaching, thus enhancing the process sustainability; good yields were realized using benzylamine under H_2_. These interesting findings were then applied to the MW-assisted one-pot synthesis of secondary amines via a tandem reductive amination of benzaldehyde with nitroaromatics under H_2_ pressure. The formation of a Cu_x_Pd_y_ alloy under reaction conditions was discerned, and a synergic effect due to the cooperation between Cu and Pd has been hypothesized. During the reaction, the system worked as a bifunctional nanocatalyst wherein the Pd sites facilitate the reduction of nitro compounds, while the Cu species promote the subsequent imine hydrogenation affording structurally diverse secondary amines with high yields.

## 1. Introduction

The synthesis of amines and their derivatives represents one of the most active themes in organic chemistry. Nitro compounds, as easily accessible starting materials, have been extensively explored for the synthesis of primary amines with productive results [1]. The as-formed primary amines can be further upgraded into diverse and highly valuable chemicals (such as secondary or tertiary amines, imines, azo compounds, and other nitrogen-containing compounds), which are useful building blocks in the synthesis of polymers, dyes, surfactants, pharmaceuticals, and agrochemicals [2,3,4,5,6,7]. Recently, the synthesis of primary amines through direct reductive amination of aldehydes using ammonia as a nitrogen source has been explored. However, the transition metal-catalyzed reductive aminations have frequently been accompanied by unwanted side product(s) due to, for example, the reduction of aldehyde to an alcohol [8,9,10]. 

The catalyst plays a key role in the reductive amination protocols, in which noble metals comprising Pd, Pt, and Rh are metals of choice due to their excellent hydrogenation and dehydrogenation effectiveness [11]. In this regard, a variety of reductive amination methods catalyzed by heterogeneous Pd or several other transition metals have been developed [12,13].

However, to improve the sustainability of the process, the development of alternative catalysts has recently received growing attention. Of late, nanocatalysis have become an emerging field of science due to its high activity, selectivity, and productivity. The nanoscale size, shape, and an exceptionally large surface area to volume ratio impart unique properties to nanocatalysts because of the structural and electronic changes, which differentiate them from the bulk materials [14].

Heterogeneous catalysis represents one of the oldest commercial practices of nanoscience; nanoparticles of metals, semiconductors, oxides, and other compounds have been widely used for important chemical reactions. Among diverse materials deployed, especially supported nanoparticles-based heterogeneous catalysts, are of interest due to their easy separation, potential activity, and selectivity [15]. Nickel nanoparticles prepared via pyrolysis of in situ generated Ni-tartaric acid complex have been recently described as efficient catalysts for the synthesis of functionalized amines starting from carbonyl compounds and ammonia in the presence of molecular hydrogen [16].

As the recyclability of the catalysts is a stringent issue in terms of sustainability, magnetic nanoparticles could represent a breakthrough in the field of green synthesis [17,18]. Among these, supported iron oxide nanocatalysts have been the focus of assorted catalytic applications because of their low cost and toxicity, ready availability, and environmentally benign nature [19]. Recently, an AuPd alloy nanocatalyst supported on Fe_3_O_4_ was employed for a one-pot reduction of a nitro compound followed by reductive amination of the resulting amine [20]. In this framework, we recently reported an efficient and heterogeneous catalytic system based on nickel silica eggshell iron-based magnetic nanoparticles (Fe_3_O_4_@SiO_2_-Ni) for a fast and quantitative microwave (MW)-assisted reductive amination of aryl aldehydes and ketones in aqueous ammonia [21]. The relentless demand for more sustainable processes requires an efficient and safer energy transfer that can be nicely achieved by MW dielectric heating; advantages related to the use of MW in organic synthesis are well documented in the literature [22]. MW promotes the remarkable reduction of reaction time, yields enhancement, and cleaner reactions compared to conventional thermal heating. MW irradiation is, therefore, a suitable tool to overcome the limitations encountered in challenging organic reactions, such as direct reductive amination in the presence of heterogeneous catalysts and gaseous reagents. Moreover, from the viewpoints of green and sustainable chemistry, it is highly attractive to adopt one-pot reaction strategies. Indeed, compared to multi-step protocols, the one-pot synthesis demonstrates higher atom efficiency by avoiding tedious purification steps and reducing the amount of discharged wastes [23].

Reductive amination, the coupling of carbonyl compounds with primary amines in the presence of a reducing reagent, has been considered one of the most efficient methods for the synthesis of secondary amines. Although the reductive amination of carbonyl compounds with primary amines has enjoyed huge success, the one-pot reductive amination directly from nitro compounds has been less developed [24,25].

Some noble metals have achieved success as active catalysts for the one-pot reductive amination under mild conditions; Pd/Ag bimetallic nanocatalyst can accomplish amination under mild conditions (room temperature) and atmospheric hydrogen pressure [26], while formic acid (FA) could be exploited as a hydrogen donor in one-pot secondary amines production utilizing Ag/Pd nanoparticles supported on mesoporous graphitic carbon nitride (mpg-C_3_N_4_/AgPd) [27]. Another one-pot reductive amination of carbonyl compounds was recently reported over *N*-doped carbon-supported cobalt nanoparticles using CO/H_2_O as an efficient hydrogen donor [28].

Herein, we report a comparative study on the use of heterogeneous nanocatalysts based on Pd (II) and Cu (I) salts embedded in a β-CD network, using either FA or H_2_ as hydrogen sources. The effect of the solvent has been illustrated for the reductive amination of benzaldehyde and acetophenone with aniline and benzylamine. Aiming for MW-assisted one-pot amination reactions, the bimetallic PdCu/CβCAΤ catalyst proved more efficient than the monometallic ones, providing high yields under milder reaction conditions using H_2_ and ethanol as the solvent. 

Transmission electron microscopy (TEM), powder X-Ray diffraction (PXRD), and diffuse reflectance UV-Visible-near infrared (DR UV–Vis-NIR) were employed to investigate the structural and electronic properties of mono- and bi-metallic nanocatalysts. The characterization revealed the presence of Cu (II) as well as Pd (0) species in the form of small copper oxide nanoparticles and metallic Pd nanoparticles in the bimetallic PdCu/CβCAΤ catalyst. During the reaction, these species are transformed to produce a Cu_x_Pd_y_ alloy, wherein both metallic sites cooperated to promote the catalytic activity enhancement.

## 2. Results

Catalytic reductive amination of carbonyl compounds with primary amines in the presence of a reducing reagent is considered one of the most efficient methods for the synthesis of secondary amines [29,30]. However, these reactions are often non-selective due to over alkylation and the reduction of carbonyl compounds to the corresponding alcohols. To circumvent these problems, the development of suitable catalytic systems that enables a fast amination reaction under milder conditions has been a challenge. In this area, a variety of reductive amination methods, catalyzed by heterogeneous Pd or several other transition metals, have been developed in recent years [31]. In view of our prior experience, we report herein the development of distinctive nanocatalysts (CβCAT) based on palladium and copper salts embedded in a β-CD network for the selective reductive amination of carbonyl compounds to secondary amines.

### 2.1. Catalyst Preparation and Characterization

In the search for more sustainable processes, nanostructured catalysts have been the subject of considerable academic and industrial research attention in recent times due to the numerous potential benefits related to their use (minimal chemical waste, and energy efficiency, among others). Despite exhibiting high catalytic activity and selectivity like homogeneous catalysis, the encapsulation of metal within a polymeric network renders the nanocatalyst recoverable, thus avoiding metal leaching [32,33]. Several metals loaded CβCAT were prepared by the reticulation of Pd/β-CD, Cu/β-CD, and PdCu/β-CD with hexamethylenediisocyanate (HDI), according to a previously documented procedure [34,35]. 

**Figure 1:**
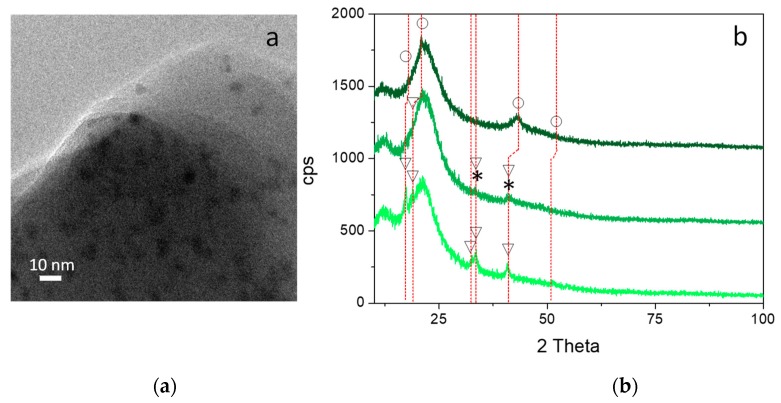
(**a**) TEM image of as-prepared Pd/CβCAT. Instrumental magnification 150,000×. (**b**) Powder X-ray diffraction (XRD) patterns of as-prepared Cu/CβCAT (light green line), as prepared PdCu/CβCAT (green line) and of PdCu/CβCAT after reaction (dark green line). The peak positions are signaled by dashed red lines. (Triangles: Copper oxide; asterisks: Metallic palladium; circles: PdCu alloy).

The structure of Pd/CβCAT has been previously investigated by using powder X-ray diffraction (PXRD), underlining the re-organization of the polymer crystalline phase into a less ordered material upon metal addition and the presence of highly dispersed Pd nanoparticles of ~3 nm that were oxidized at the surface (owing to exposure to air) [36]. A representative TEM image of the as-prepared Pd/CβCAT catalyst is depicted in Figure 1a. Round Pd nanoparticles well contrasted with respect to the polymeric support and of size ranging between 3 and 5 nm were clearly detected, in agreement with the previous PXRD results. Notably, the Pd/β-CD fresh sample proved to be stable with prolonged exposure to the electron beam of the instrument, since neither metal coalescence nor polymer degradation was detected. 

The comparative PXRD patterns for the Cu/CβCAT and PdCu/CβCAT are presented in Figure 1b. In particular, the patterns displayed a broad peak that was related to the presence of crosslinked hexamethylene diisocyanate amorphous moieties [36], along with with less intense peaks in the 15 to 50 2Theta range that revealed the presence of highly dispersed metal nanoparticles. 

As for Cu/CβCAT (light green line), the peaks centered at 16.9°, 18.9°, 32.6°, 33.4°, 40.9°, and 51.1° could be related to Cu-rich orthorhombic copper oxide (Cu_64_O, JCPDS file number 01-077-1898). These peaks were observed with lower intensity in the case of as-prepared PdCu/CβCAT (green line) along with the presence of components at 2Theta 40.4° and 46.1°, due to metallic Pd in the cubic phase (JCPDS file number 00-001-1201). The latter features were indicative of the presence of a reduced Pd phase on the bimetallic catalyst, otherwise observed for the monometallic Pd/CβCAT [36].

**Figure 2:**
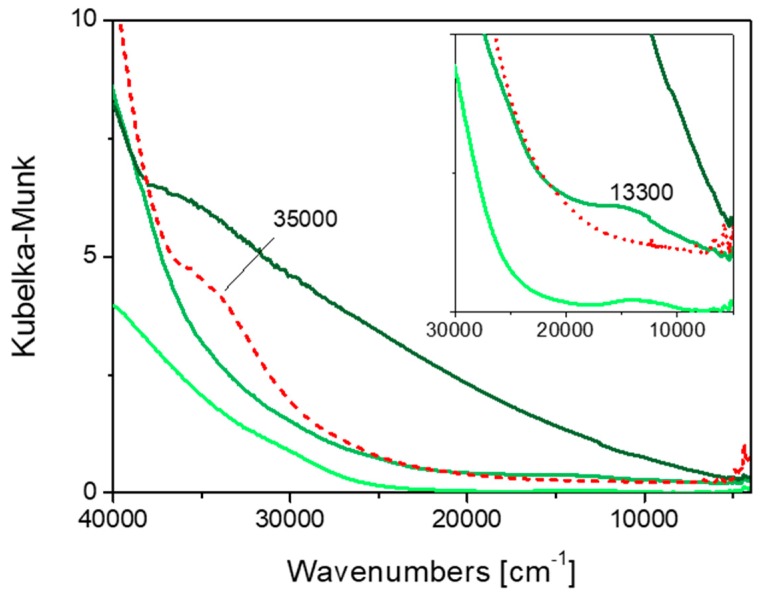
DR UV-Vis_NIR spectra of as-prepared Cu/CβCAT (light green line), as prepared PdCu/CβCAT (green line) and of PdCu/CβCAT after reaction (dark green line). The spectrum of crosslinked βCD (red dashed line) is also reported for comparison. Inset: Spectra shown in the 30,000 to 5000 cm^−1^ range.

DR UV–Vis-NIR characterization was carried out on Cu/CβCAT (light green line), as-prepared PdCu/CβCAT (green line), and the results are reported in Figure 2. Diffuse reflectance UV-Vis spectroscopy was employed to gain information about the Pd and Cu oxidation states. Especially, a band at 24,000 cm^−1^, related to the d-d transition of Pd (II) species, was found on PdCu/CβCAT (data not shown). This indicates that this catalyst contained highly dispersed PdO particles, in agreement with previous PXRD findings [36].

A rather intense absorption band at 35,000 cm^−1^ attributed to the π−π * electronic transition from the HOMO to LUMO of crosslinked βCD [37] (red dashed line) was depleted upon metal insertion, a weak absorption, assigned to the d-d transition in Cu (II) species [38,39] was observed at about 13,300 cm^−1^ on both Cu/CβCAT (inset, light green line) and as-prepared PdCu/CβCAT (inset, green line).

### 2.2. Reductive Amination

Aiming for the development of a sustainable reductive amination protocol, we combined the advantages of heterogeneous catalysts, such as the potential recyclability and negligible metal leaching and those emanating from non-conventional technologies. The multimode MW reactor used for the reductive amination was equipped with a pressure control system, and a multiple position rack, suitable for multiple gas loading (both inert and reactive one) and parallel reaction runs. Easy reaction scale-up (up to 10 mol scale) could also be achieved in the same reactor using different reaction vessels (maximum 1 L capability). The use of two reducing agents was exploited and compared for this purpose: H_2_ as an environmentally friendly reducing agent (water was the only generated oxidation by-product), and FA. This latter H-donor has been regarded as a promising hydrogen storage material owing to its advantageous features (e.g., high volumetric hydrogen capacity (53 g L^−1^); liquid at room temperature, non-toxicity, and low cost) [40].

**Scheme 1:**
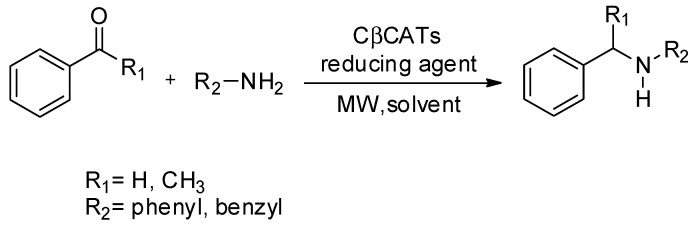
CβCATs reductive amination.

Benzaldehyde and acetophenone were used as model substrates to test the MW-assisted reductive aminations with aniline or benzylamine over three different nano-CβCATs: Cu/CβCAT, Pd/CβCAT, and PdCu/CβCAT. (Scheme 1, Table 1).

**Figure 3:**
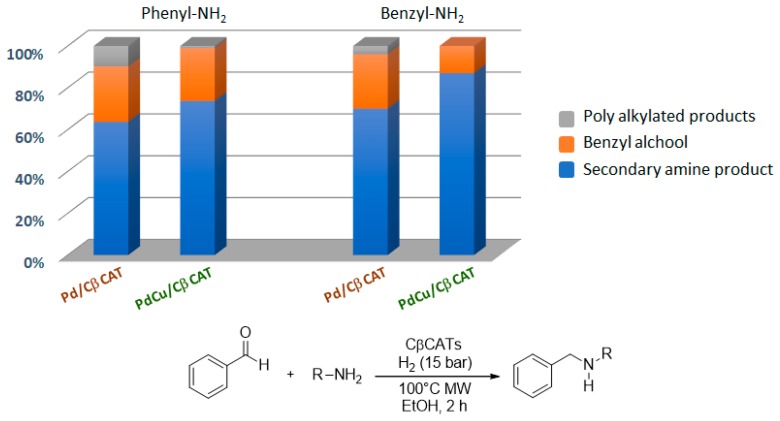
Influence of the co-metal on the bimetallic catalyst performance and selectivity.

The MW-assisted reductive aminations were performed first at 100 °C on a 0.5 mmol scale adopting a catalyst loading of 5 wt % (referred to the metal content of CβCATs) in the presence of H_2_ (15 bar) or FA (4 eq); either ethanol or water were exploited as sustainable solvents for this purpose. Using PdCu/CβCAT under H_2_ pressure, practically good results were obtained for the reductive amination of benzaldehyde with either aniline or benzylamine. In particular, benzylphenyl amine and dibenzylamine were obtained in excellent (73% and 87%) and good (62% and 78%) yields in ethanol and water, respectively, as the only reaction products (Table 1, entries 3 and 9), thus avoiding the direct benzaldehyde hydrogenation over PdCu/CβCAT. Gratifyingly, when reactions were performed under H_2_ pressure, the PdCu/CβCAT exhibited fairly good activity and selectivity towards the reductive amination of ketones as well (Table 1, entries 6 and 12). Especially, the aliphatic amine afforded up to 80% of *N*-benzyl-1-phenylethanamine in ethanol, whereas only 49% was achieved in water (Table 1, entry 12). The monometallic catalysts were not so effective; for example, Cu/CβCAT proved to be completely inactive for the reductive amination of both benzaldehyde and acetophenone at 100 °C under H_2_ either in ethanol or in aqueous solutions (Table 1, entries 1, 4, 7, and 10). Conversely, Pd/CβCAT afforded the secondary amines in good yields only when benzaldehyde was reacted under MW for 2 h, with better results in ethanol for both benzyl phenyl amine (65% vs. 57%) and dibenzylamine (70% vs. 64%) (Table 1, entries 2 and 8). Yet, this catalyst showed no activity in the reductive amination of acetophenone. The influence of the co-metal on the catalyst performance in bimetallic palladium catalysts (the alloying effect) is known [41]. Importantly, the introduction of another metal (Cu) into the intrinsically active Pd/CβCAT led to a catalyst of fine-tuned activity, improving the selectivity of the reaction [26]. In contrast, benzyl alcohol, polyalkylated compounds were detected as main by-products when using the monometallic Pd nanocatalyst (Figure 3).

To further improve the product yields, we studied the reductive amination at elevated temperature (180 °C). The same catalyst activity trend was maintained (PdCu/CβCAT > Pd/CβCAT >>> Cu/CβCAT), but unfortunately, no better results were recorded. However, the noticeable fact was that PdCu/CβCAT enabled higher product yields in water rather than in ethanol, reversing the previously encountered behavior at 100 °C (Table 1).

FA was subsequently tested as a hydrogen donor for MW assisted reductive aminations because it represents an economical, safe, and straightforward way for the in-situ generation of hydrogen. The catalytic activity of the three different CβCATs in the presence of 4 equiv. of FA (lower amounts of FA could lead to reduced yields [29]) were compared using the same benchmark reaction and conditions (2 h MW irradiation at 100 °C or 180 °C) adopted for reductive amination under H_2_ pressure (Table 1). However, as part of the amine was consumed by FA to form the corresponding formamide as a by-product, more amine (4 equiv.) was added as a sacrificial component to improve the yields of the desired products. As is well documented in the literature, Pd-based catalysts suffer from deactivation problems during FA dehydrogenation owing to the strong adsorption of formate anions [27]. To overcome this deactivation difficulty, Pd has been recently combined with various metals including Au, Ag, and Ni, in a bimetallic or a multimetallic alloy or a core–shell structure [42]. Herein, we combined Pd with Cu into a β-CD-network to generate a more active PdCu/CβCAT. Actually, this bimetallic catalytic system enabled higher reductive amination yields than a monometallic one in the presence of FA (Table 1).

However, although the increased activity of PdCu/CAT was maintained while using FA rather than H_2_ gas, lower yields were attained for benzaldehyde and acetophenone reductive amination over both Pd/CβCAT and PdCu/CβCAT at 100 °C (Table 1). Moreover, using FA, no significant differences in reaction yields were observed, changing the reaction solvent (water or ethanol), and a large amount of formamide was detected as the by-product. An attempt at a higher temperature (180 °C) failed to increase the yield. However, it must be emphasized that unlike what was observed under external hydrogen pressure, the present reductive amination protocol enabled benzaldehyde conversion in the presence of both aniline and benzylamine when Cu/CβCAT was used. As is well-known, the surface structure and oxidation state of the metal may play a role in selectivity [43].

Therefore, according to the characterization results, such behavior can be due to the presence of Cu-rich copper oxide species within the crosslinked βCD matrix. In order to be effective, the Cu (II) species would have to be converted, possibly through the formation of Cu (I) species, into active Cu (0) species to enable the activation of the hydrogen molecule. This could explain the lack of activity under H_2_ pressure because Cu (II) species did not activate hydrogen. Conversely, the catalytic transfer hydrogenation occurred when the reaction mixture containing Cu/CβCAT was irradiated in the presence of FA. 

As a result of the optimization experiments, it was found that the best reductive amination yields for both aldehyde and ketone moieties can be acquired under MW irradiation by using the described bimetallic PdCu/CβCAT in ethanol at 100 °C for 120 min. under H_2_ pressure (15 bar).

**Scheme 2:**
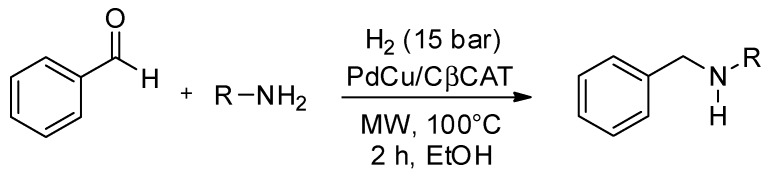
CβCATs reductive amination reaction.

The general applicability of this bimetallic catalyst for the synthesis of secondary amines under the optimum reaction conditions (100 °C, 2 h, H_2_ pressure, EtOH) (Scheme 2) was then evaluated. The results about the MW-assisted reductive amination of benzaldehyde with assorted aryl- and alkyl-amines are summarized in Table 2. 

Based on the reported results, it is worth noting that all varieties of deployed substrates successfully underwent reductive aminations; good and comparable yields (>64%) were achieved from the reductive amination of aromatic aniline and 4-methylaniline with benzaldehyde to corresponding secondary amines (Table 2, entries 1 and 2). Although the longer reaction time was required (up to 2.5 h, yield 82%), the efficient reductive amination of 4-methoxyaniline (Table 2, entry 3) was observed. The electron-donating effect of 4-methoxy- substituent on aniline could stabilize the imine intermediate requiring a longer time for the reduction. Analogous behavior was observed for 4-iodoaniline to reach 61% yield of desired secondary amine (Table 2, entry 4); a slightly lower yield was detected for 4-trifluoromethylaniline (57%) (Table 2, entry 5). Superior results (87% yield) were recorded for benzylamine (Table 2, entry 6), and even more hindered β-methyl substituted benzylamine afforded good yields (76%) (Table 2, entry 7). Ultimately, the reactions of alkyl amines such as octylamine and 2-methylpropan-1-amine proceeded quickly to produce the corresponding alkyl benzylamines in excellent yields (Table 2, entries 8 and 9).

### 2.3. Reduction of Nitroaromatics

**Scheme 3:**
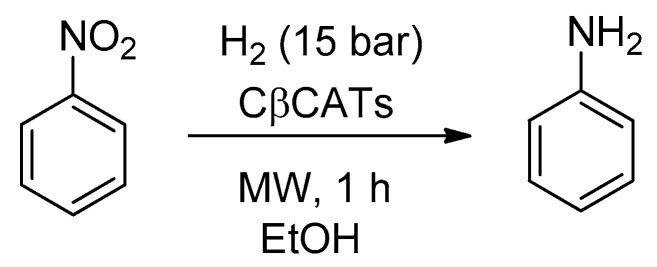
MW assisted reduction of nitroaromatics in the presence of CβCATs.

The reduction of nitrobenzene was used as a benchmark reaction (Scheme 3) to optimize the conditions for the reduction of the nitro group in the following one-pot reactions. The reaction was performed in EtOH at 100 °C, as these were the optimized conditions for reductive amination.

Quite surprisingly, using the bimetallic nanocatalyst at 100 °C, the nitrobenzene was recovered almost unreacted (1% of aniline was detected, Table 3), while the highest yield was realized using Pd/CβCAT (87.5%). As PdCu/CβCAT was the best performing catalyst for the reductive amination, it was tested for the reduction of nitrobenzene at higher temperatures. An experiment at 150 °C afforded 68% of aniline; a further increase of temperature was not taken into consideration since it could negatively impact the reductive amination step, as described earlier (see the previous section). On the contrary, dicyclohexylamine was the main product (58%) formed when Pd/CβCAT was used at 150 °C. As already observed in the reductive aminations, the monometallic Cu/CβCAT was completely inactive, owing to the presence of Cu(II) species.

### 2.4. One-Pot Reductive Amination

**Scheme 4:**
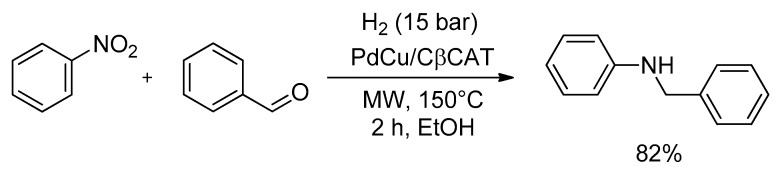
One-pot reductive amination.

Once the optimum conditions have been determined, we set out to evaluate the catalytic activity of the bimetallic catalyst using benzaldehyde and nitrobenzene as the model system for the one-pot reductive aminations (Scheme 4).

The best yield of *N*-benzylaniline was achieved at 150 °C, while working at 100 °C there was no product formation detected. Product yields were dramatically reduced when the temperature was enhanced to 180 °C, as only 34% *N*-benzylaniline was obtained. Interestingly, the position of the peaks observed on the PdCu/CβCAT after the one-pot amination reaction (see Figure 1, dark green line) was shifted toward high angles with respect to that of the as-prepared catalyst (green line). Peaks at 2Theta 17.8°, 21.0°, 43.0°, and 52.0° may be ascribed to a copper-rich Cu_x_Pd_y_ alloy in the tetragonal phase (Cu_2.991_Pd_1.009_, JCPDS file number: 03-065-9675). DR UV–Vis-NIR characterization (Figure 2) revealed quite big differences between the spectra of the bimetallic catalyst before (inset, green line) and after (inset, dark green line) the reaction. In particular, an exponentially increasing absorbance towards higher energy was observed as a consequence of interband transitions [44]. Such spectroscopic feature can be reasonably related to the formation of bimetallic particles under reaction conditions, in agreement with PXRD analysis. Therefore, we can hypothesize a synergic effect due to the formation of a Pd_x_Cu_y_ alloy, where the Pd sites facilitate the reduction of the nitro compound, whereas the Cu species could promote the imine hydrogenation to secondary amine.

Negligible metal leaching was observed after completion of the reaction; indeed, after ICP-OES analysis, the metal content of 87.10 ± 1.35 g/kg of Cu and 84.98 ± 1.57 g/kg of Pd were detected in the PdCu/CβCAT.

## 3. Materials and Methods

All starting organic reagents and solvents were purchased from Sigma Aldrich (Sigma Aldrich, Darmstadt, Germany) and used without further purification. The β-CD was purchased from Wacker Chemie, Munich, Germany, and MW-assisted reactions were carried out in a SynthWAVE reactor (MLS GmbH, Leutkirch im Allgäu, Germany; Milestone Srl, Bergamo, Italy), which houses a closed MW-cavity. The GC-MS analyses were performed with an Agilent 6890 system (Agilent Technologies, Santa Clara, CA, USA) fitted with a mass detector Agilent Network 5973 using a capillary column that was 30 m long and had an i.d. of 0.25 mm and a film thickness of 0.25 mm. GC conditions were: Injection split of 1:20, injector temperature of 250 °C, and detector temperature of 280 °C. The gas carrier was helium (1.2 mL min^−1^), and the temperature program was from 70 °C (2 min) to 300 °C at 5 °C min^−1^. The CβCAT metals content was determined using ICP-OES with a Perkin Elmer Optima 7000 (Perkin Elmer, Norwalk, CT, USA) spectrometer via the addition of a standard. 

Transmission electron microscopy (TEM) measurements were carried out by employing a side entry Jeol JEM 3010 (300 kV) microscope equipped with a LaB_6_ filament and fitted with X-ray EDS analysis by a Link ISIS 200 detector. The powdered sample was deposited on a Cu grid, coated with a porous carbon film, and the digital micrographs were acquired using an Ultrascan 1000 camera. The images were processed by Gatan digital micrograph. 

Powder X-ray diffraction (PXRD) patterns were collected with a PW3050/60 X’Pert PRO MPD diffractometer from PANalytical working in Bragg–Brentano geometry, using as a source the high-powered ceramic tube PW3373/10 LFF with a Cu anode (λ = 0.541 Å) equipped with a Ni filter to attenuate *K*_β_. Scattered photons were collected by a real-time multiple strip (RTMS) X’celerator detector. Data were collected in the 5° ≤ 2*θ* ≤ 100° angular range, with 0.02° 2*θ* steps. 

Diffuse reflectance (DR) UV–Vis-NIR spectra were recorded at room temperature on a Varian Cary 5000 spectrophotometer equipped with an integrating sphere attachment (BaSO_4_ as internal reference), in the 50,000 to 4000 cm^−1^ range. DRUV–Vis-NIR spectra of the as-prepared samples were reported in the Kubelka-Munk function [f (R_∞_) = (1 − R_∞_)^2^/2R_∞_; R_∞_ = reflectance of an “infinitely thick” layer of the sample.

### 3.1. PdCu/β-CD Catalyst Preparation 

An easy and reproducible one-pot sonochemical β-CDs reticulation in the presence of hexamethylene diisocyanate (HDI) and Pd(II) or Cu(I) salts has been employed to obtain Pd/CβCAT, Cu/CβCAT, and PdCu/CβCAT [34]. To this end, the HDI crosslinking agent (2.8 mL, 17.4 mmol) was slowly added under intense sonication to a β-CD (1 g, 0.78 mmol) solution in DMF (4 mL) together with Pd(OAc)_2_ (200 mg, 0.90 mmol) or CuCl (90 mg, 0.9 mmol). For the preparation of the bimetallic catalyst, 0.45 mmol (100 mg) of Pd(OAc)_2_ and 0.45 mmol (45 mg) of CuCl were added. The reaction mixture was thermostated at 60 °C, and after 30 min sonication (20 kHz), a compact gel was obtained. This reticulated product was crushed and washed twice with water, methanol, and acetone (50 mL each). The final CβCAT was filtered on a sintered glass Buchner funnel and dried overnight under vacuum at 75 °C, to produce after 20 min grinding at 650 rpm in ball mill (Retsch PM100 High Speed) a dark green powder (PdCu/CβCAT), a brownish powder (Pd/CβCAT), and a light green powder (Cu/CβCAT), respectively. The particle size of the catalyst powder, measured by photon correlation spectroscopy (Coulter) after dispersion in water, was found to be in the range of 700 to 1100 nm.

The CβCATs were then analyzed by ICP-OES, revealing an average metal content of 89.10 ± 1.43 g/kg (Cu/CβCAT) and 88.90 ± 1.58 g/kg (Pd/CβCAT). In the bimetallic catalyst 87.32 ± 1.55 g/kg of Cu and 85.13 ± 1.71 g/kg of Pd were detected.

### 3.2. Microwave-Assisted Reductive Amination

MW-promoted reactions were carried out in the SynthWAVE reactor, a multimode system that enables multiple gas inlet. This instrument equipped with a high-pressure stainless-steel reaction chamber, which can work up to a maximum of 300 °C temperature and 199 bar, enables MW reactions both in small-scale (mL) and large-scale (L). Moreover, integrated reactor sensors continuously monitored the internal pressure, temperature, and power applied inside the reactor cavity during all reaction runs, adjusting the applied MW power in real-time to follow a predefined temperature profile. A sampling valve on the bottom of the reactor was directly connected to the cooling chamber.

In a typical experiment, benzaldehyde or acetophenone (0.5 mmol) were reacted with amine in 2 mL of solvent (EtOH or H_2_O) in the presence of 94 mg of PdCu/CβCAT, corresponding to the 5 wt % of metal (Pd content = wt %, Cu content = wt % determined by ICP-OES). The reactions were performed under magnetic stirring (450 rpm) at fixed temperature and time (see Table 1) in an MW SynthWAVE reactor that enables multiple gas inlet.

#### 3.2.1. Reductive Amination under H_2_ Pressure

When H_2_ was used for reductive amination, 0.5 mL amine was added to benzaldehyde or acetophenone and, after flushing the MW reactor 3 times with N_2_, a pressure of H_2_ (15 bar) was loaded at room temperature before starting the heating program. 

#### 3.2.2. Reductive Amination in the Presence of Formic Acid

When the reductive amination was performed in the presence of an H-donor, FA (76 µL, 2 mmol), the amine (2 mmol) was added to the reaction mixture before starting the heating program and loading the MW reactor chamber with N_2_ (15 bar).

Upon completion of the heating stage, the reaction chamber was cooled down to room temperature. The catalyst PdCu/CβCAT was removed by filtration and washed twice with ethanol. Then, 100 µL of the reaction mixture was diluted with CHCl_3_ (1 mL), and the resulting solution was analyzed by GC-MS. 

When water was used as a reaction solvent, the products were extracted with CHCl_3_ (2 × 2 mL) and, after drying over Na_2_SO_4_, the organic phase was injected into GC-MS. The GC analyses were performed with an Agilent 6890 system (Agilent Technologies, Santa Clara, CA, USA) fitted with a mass detector Agilent Network 5973 using a capillary column that was 30 m long and had an i.d. of 0.25 mm and a film thickness of 0.25 mm. GC conditions were: Injection split of 1:20, injector temperature of 250 °C, and detector temperature of 280 °C. The gas carrier was helium (1.2 mL min^−1^), and the temperature program was from 70 °C (2 min) to 300 °C at 5 °C min^−1^. 

### 3.3. Microwave-Assisted Reduction of Nitrobenzene

In a typical experiment, nitrobenzene (0.5 mmol) was dissolved in 2 mL of ethanol in a flask. After the addition of the catalyst (5 mol% of metal), the mixture was stirred (450 rpm) at the selected temperature under H_2_ atmosphere (15 bar) for 1 h in SynthWave. After cooling, the crude reaction was filtered, and 100 µL of the reaction mixture was diluted with CHCl_3_ (1 mL) and analyzed using GC-MS (Agilent Technologies, Santa Clara, CA, USA).

## 4. Conclusions

The development of new heterogeneous catalysts for organic transformations is a significant task for modern synthetic chemistry. In this respect, herein, we report the use of new nanocatalytic systems for the synthesis of secondary amines from nitroarenes via a one-pot MW-assisted reductive amination of carbonyl compounds under H_2_ pressure. The bimetallic PdCu/CβCAT catalyst showed fine-tuned activity, due to the formation under reaction conditions, of a Cu_x_Pd_y_ alloy, in which the metallic sites can synergistically afford the best results in terms of yields and selectivity, without any metal leaching. Thus, it can be suggested that the PdCu/CβCAT system could work as a bifunctional nanocatalyst, in which the Pd sites could facilitate the reduction of nitro compounds, affording structurally diverse secondary amines in high yields. The role of copper is still under investigation to shed light on the imine reduction step. The synthesis of secondary amines directly starting from nitroaromatics as precursors is advantageous for synthetic organic chemistry in terms of both step economy and cost of the substrates. The present catalytic reductive amination protocol has several advantages over the existing methodologies in the literature, such as providing the atom economy by the elimination of extra reaction steps along with the high product yields in reduced times. The use of a bimetallic nanocatalyst combines the advantages coming from both homogeneous (for selectivity and high yields) and heterogeneous catalysis (for easy catalyst recovery and no metal leaching).

## Figures and Tables

**Table 1:** MW-Assisted Reductive Amination (CβCAT comparison).

				Yield (%) ^c^							
				H_2_ (_15 bar_)				FA (_4 equiv._) ^d^			
Entry ^a^	Substrates		Catalyst	100 °C		180 °C		100 °C		180 °C	
	R_1_	R_2_^b^		EtOH	H_2_O	EtOH	H_2_O	EtOH	H_2_O	EtOH	H_2_O
1	H	Phenyl	Cu/CβCAT	0	0	0	4	45	46	28	25
2			Pd/CβCAT	65	57	45	50	47	48	39	32
3			PdCu/CβCAT	73	62	54	67	53	53	54	42
4	CH_3_		Cu/CβCAT	0	0	0	0	0	0	2	0
5			Pd/CβCAT	0	0	3	0	15	12	14	11
6			PdCu/CβCAT	24	17	13	19	37	34	22	20
7	H	Benzyl	Cu/CβCAT	0	0	0	19	55	46	33	32
8			Pd/CβCAT	70	64	0	71	53	52	47	40
9			PdCu/CβCAT	87	78	48	80	75	76	59	64
10	CH_3_		Cu/CβCAT	0	0	0	0	4	0	2	8
11			Pd/CβCAT	0	24	0	0	38	30	23	21
12			PdCu/CβCAT	80	69	58	63	61	53	39	37

^a^ Reactions performed under MW irradiation on a 0.5 mmol scale of aldehyde or ketone, 1 equiv. of amine in the presence of CβCAT (5 wt % metal loading) under H_2_ pressure (15 bar) into 2 mL solvent (ethanol or water); ^b^ 4 equiv. of amine was used in the presence of FA (4 equiv.); ^c^ Determined by GC/MS ^d^ N_2_ back pressure (15 bar).

**Table 2:** PdCu/CβCAT catalyzed the reductive amination of different amines with benzaldehyde.

Entry ^a^	R-NH_2_	Yield (%) ^b^
1	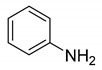	73
2	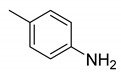	64
3	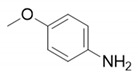	82 ^c^
4	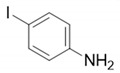	61 ^c^
5	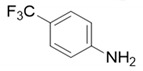	57
6	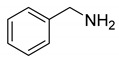	87
7	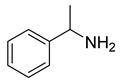	76
8	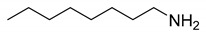	84
9	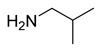	68

^a^ Reaction conditions: 0.5 mmol benzaldehyde, 0.5 mmol amines, 5 wt % PdCu/CβCAT catalyst (based on Pd), 2 mL ethanol, MW irradiation at 100 °C for 2 h, H_2_ (15 bar); ^b^ Determined by GC-MS (yields are based on area percentage); ^c^ Reaction time 2.5 h.

**Table 3:** MW assisted Nitroarene reduction tests in the presence of CβCATs ^a^.

Catalyst	Yield (%) ^b^	
	100 °C	150 °C
Cu/CβCAT	0	0
Pd/CβCAT	87.5	17
PdCu/CβCAT	1	68

^a^ Reaction conditions: 0.5 mmol nitrobenzene, 5 wt % CβCAT (based on metal), 2 mL ethanol, MW irradiation at 100 °C or 150 °C for 1 h, H_2_ (15 bar); ^b^ Determined by GC-MS (yields are based on area percentage).
